# Sleep discrepancy and cognitive function in community‐dwelling older adults

**DOI:** 10.1111/jsr.14288

**Published:** 2024-07-26

**Authors:** Nadia Soh, Stephanie R. Rainey‐Smith, James D. Doecke, Rodrigo Canovas, Romola S. Bucks, Melissa Ree, Michael Weinborn

**Affiliations:** ^1^ School of Psychological Science University of Western Australia Crawley Western Australia Australia; ^2^ Centre for Healthy Ageing, Health Futures Institute Murdoch University Murdoch Western Australia Australia; ^3^ Australian Alzheimer's Research Foundation Sarich Neuroscience Research Institute Nedlands Western Australia Australia; ^4^ School of Medical and Health Sciences Edith Cowan University Joondalup Western Australia Australia; ^5^ Australian E‐Health Research Centre CSIRO Herston Queensland Australia; ^6^ Australian E‐Health Research Centre CSIRO Melbourne Victoria Australia; ^7^ School of Population and Global Health University of Western Australia Crawley Western Australia Australia

**Keywords:** ageing, cognitive function, insomnia, sleep discrepancy

## Abstract

This was the first study to use cluster analysis to characterise sleep discrepancy (the discordance between self‐reported and objective sleep) across multiple sleep parameters, in community‐dwelling older adults. For sleep efficiency, negative discrepancy (the tendency to self‐report worse sleep than objectively‐measured) was associated with poorer memory, independent of insomnia severity, depressive symptoms and objective sleep. This suggests a unique role for sleep discrepancy as a possible risk factor for future cognitive decline, and warrants the need for further research.

## Graphical abstract

In older adults, distinct sleep discrepancy groups were identified through cluster analysis (“positive”, “negative”, and “no” sleep discrepancy). Negative discrepancy across two sleep parameters (wake after sleep onset and sleep efficiency) was associated with poorer delayed memory.

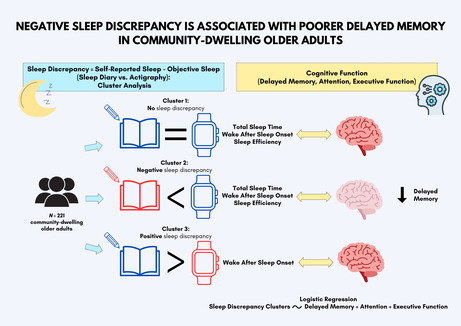

## 1 INTRODUCTION

Approximately 50% of older adults (e.g. 55+ years) complain of sleep disturbances (Foley et al., 1995). This is salient, given the link between suboptimal sleep and adverse health outcomes, including poorer cognitive function (Dzierzewski et al., 2018). Interestingly, a relationship between suboptimal sleep and poorer cognition has been found with both self‐report (e.g. questionnaires) and objective (e.g. polysomnography) sleep measures (Dzierzewski et al., 2018), despite evidence that individuals are often “inaccurate” in their sleep perception. That is, research has often found a discordance between self‐report and objective sleep measures, referred to as sleep discrepancy (Van Den Berg et al., 2008).

Sleep discrepancy shows promise as a clinical tool, with research linking greater discordance between self‐report versus objective sleep to poorer cognition (Van Den Berg et al., 2008), adverse physical and mental health outcomes (Jackowska et al., 2011), and greater all‐cause mortality (Utsumi et al., 2022). However, the consequences of sleep discrepancy are not well‐defined for older adults, where discrepancy (Van Den Berg et al., 2008), as well as poor sleep and cognitive decline, are of increased prevalence (Dzierzewski et al., 2018). The current study aimed to address this gap by evaluating patterns of sleep discrepancy and their relationship to cognition in community‐dwelling older adults.

Sleep discrepancy is characterised by both magnitude and direction, and is typically operationalised using a difference score (i.e. sleep discrepancy = self‐report sleep – objective sleep), although there are variations in methodology (Manconi et al., 2010). Discrepancy may be evaluated across multiple sleep parameters, including total sleep time (TST), sleep‐onset latency (SOL; i.e. time to transition from wake to sleep), number of awakenings (NWAK), wake after sleep onset (WASO; i.e. time awake after falling asleep) and sleep efficiency (SE; i.e., ratio of TST to time in bed [TIB]; Most et al., 2012). “Negative discrepancy”, self‐reporting worse sleep than objectively‐measured, may represent a tendency towards overestimating sleep problems, and is linked to conditions characterised by a negative information‐processing bias such as insomnia and depression (Rezaie et al., 2018; Van Den Berg et al., 2008). Conversely, “positive discrepancy” describes the opposite pattern. For example, individuals with cognitive impairment may display a positive discrepancy (Most et al., 2012), perhaps due to lack of awareness causing inaccurate recall of sleep. Interestingly, however, positive discrepancy has also been observed in insomnia and depression (Rotenberg et al., 2000; Yoon et al., 2022), which may reflect the heterogeneity of these conditions.

In older adults, sleep‐related effects on cognition are of particular interest, given that they may work alongside other age‐related processes affecting cognition. Findings in the context of sleep discrepancy, however, are less established, although some relationships with cognition have emerged. There are relatively consistent findings of sleep discrepancy in mild cognitive impairment and Alzheimer's disease, suggesting a link between discrepancy and cognitive decline. However, the direction of this discrepancy is less clear; that is, both negative (Hita‐Yanez et al., 2013) and positive (DiNapoli et al., 2017; Most et al., 2012) discrepancy have been found. Conversely, research with cognitively unimpaired community‐dwelling older adults has been more mixed; absolute (Van Den Berg et al., 2008; Winer et al., 2021), negative (Winer et al., 2021) and positive (Van Den Berg et al., 2008) discrepancy have been associated with poorer cognition, although other studies have found no association (Baillet et al., 2016).

Although previous research provides intriguing initial findings, there were limitations in the methodological approaches employed to evaluate sleep discrepancy (e.g. choice of self‐report and objective measures, and single versus multiple nights of data) and cognition (e.g. global cognition versus comprehensive assessment, and group comparison of clinical versus non‐clinical samples). Additionally, research exploring this relationship should consider the influence of related constructs such as insomnia and depression, which are associated with negative (Rezaie et al., 2018; Van Den Berg et al., 2008) and positive (Rotenberg et al., 2000; Yoon et al., 2022) discrepancy, as well as poorer cognition (McDermott & Ebmeier, 2009; Wardle‐Pinkston et al., 2019). These conditions have high comorbidity and are common in older adults (Staner, 2010). Given these overlaps, research is needed to evaluate sleep discrepancy and its relationship to cognition, with consideration for the contribution of insomnia and depression. Doing so would allow for greater understanding of sleep discrepancy, and its unique role and clinical potential, in the context of cognitive function.

The present study investigated sleep discrepancy and cognition in cognitively unimpaired community‐dwelling older adults, using cluster analysis to characterise patterns of sleep discrepancy. To the authors' knowledge, this is the first study to do so. The methodology of clustering participants into discrepancy groups was chosen given the lack of consensus on what difference score cut‐off constitutes a “meaningful” sleep discrepancy. This approach allows for characterisation of both discrepancy magnitude and direction, and is informed by both self‐report and objective sleep, which has further advantage over difference scores (Shanock et al., 2010). We evaluated discrepancy across several nights using sleep diary and actigraphy, for multiple parameters (i.e. TST, WASO and SE). Measures of cognition focusing on memory, attention and executive function, domains that have most consistently been linked to suboptimal sleep (Dzierzewski et al., 2018), were used.

Taking an exploratory approach, we assessed whether: (1) participants would form clusters characterised by differences in self‐reported sleep, objective sleep and/or sleep discrepancy across sleep parameters (i.e. TST, WASO and SE); (2) clusters characterised by sleep discrepancy (i.e. negative or positive) and/or poor sleep (i.e. self‐reported versus objective) would be associated with poorer memory, attention and/or executive function; and (3) there is a unique relationship between sleep discrepancy and cognition after controlling for insomnia severity and depressive symptoms.

## 2 METHOD

### 2.1 Participants

The current study used data from the Healthy Ageing Research Program (HARP), an observational study of thinking skills and daily functioning in community‐dwelling older adults, conducted at the University of Western Australia (UWA; *N* = 268). We included participants involved in a sleep sub‐study within HARP (*N* = 237). Participants were recruited in several ways, including flyers and word‐of‐mouth. Data were collected in 2014–2018. Study approval was obtained from the UWA Human Research Ethics Office (2021/ET000261).

Exclusion criteria included: significant psychiatric (e.g. schizophrenia; *n* = 2) or neurological (e.g. stroke; *n* = 6) disorders that could impact cognition; previous loss of consciousness for > 30 min (*n* = 6); and less than five valid nights of concurrent sleep diary and actigraphy data (*n* = 4; Littner et al., 2003). We did not implement exclusion based on scores suggestive of cognitive impairment (e.g. Mini Mental State Examination < 24; *n* = 3), in an effort to increase generalisability to community‐dwelling older adults; however, no participants met criteria for dementia. The final sample consisted of *N* = 221 participants aged 55–93 years. Sample characteristics are shown in Table 1.

**TABLE 1:** Sample characteristics.

Variable	Mean (SD) or *N* (%)	Range
Age, years	71.90 (7.50)	55–93
Sex, *N* = female (%)	140 (63.35)	–
Education, years	13.82 (3.19)	3–20
GAD‐7 total score	1.76 (2.66)	0–16
PHQ‐9 total score	2.57 (2.82)	0–13
BMI, kg m^−2^	26.30 (4.26)	18.20–49.95
Berlin risk score, *N* = low OSA risk (%)	150 (67.87)	–
ISI total score	7.62 (6.46)	0–26
Sleep diary		
TST, min	401.99 (62.89)	157.50–558.57
WASO, min	30.94 (32.30)	0.21–244.71
SE, %	79.00 (11.28)	28.08–100.00
Actigraphy		
TST, min	413.98 (50.86)	242.17–597.77
WASO, min	40.93 (21.06)	4.57–128.00
SE, %	90.24 (4.54)	70.41–98.52
Sleep discrepancy^a^		
TST, min	−12.20 (52.11)	−316.47 – 115.29
WASO, min	9.99 (34.37)	−200.71 – 116.57
SE, %	−11.24 (12.06)	−65.56 – 15.57
RBANS attention index	106.88 (14.57)	72–142
RBANS delayed memory index	101.56 (12.55)	60–126
EF composite	0.00 (1.00)	−3.21 – 2.65
WAIS‐III digit span backwards	7.29 (2.23)	2–14
TMT‐B, s	76.34 (32.20)	33.80–241.00
NIH‐EXAMINER flanker	8.32 (0.45)	6.85–9.21
COWAT‐letter fluency	16.54 (4.75)	4–33
MMSE total score	27.94 (1.56)	22–30

*Note*: *N* = 221.

Abbreviations: Berlin, Berlin Questionnaire, measure of OSA risk; BMI, body mass index; COWAT‐letter fluency, letter fluency from the Controlled Oral Word Association Test, where higher scores indicate better generativity; EF composite, executive function composite comprised of tests measuring working memory, set‐shifting, response inhibition and generativity; GAD‐7, Generalised Anxiety Disorder, where higher scores indicate greater anxiety symptoms; ISI, Insomnia Severity Index, where higher scores indicate greater insomnia severity; MMSE, Mini Mental State Examination, where higher scores indicate greater global cognitive function; NIH‐EXAMINER flanker, flanker task from the National Institutes of Health Executive Abilities: Measures and Instruments for Neurobehavioural Evaluation and Research, where higher scores indicate greater response inhibition; OSA, obstructive sleep apnea; PHQ‐9, Patient Health Questionnaire, where higher scores indicate greater depressive symptoms; RBANS, Repeatable Battery for the Assessment of Neuropsychological Status, where higher Attention and Delayed Memory Index scores suggest greater attention and delayed memory ability, respectively; SE, sleep efficiency, ratio of total sleep time to time in bed (%); TMT‐B, Trail Making Test – Part B, where lower scores indicate better set‐shifting ability (s); TST, total sleep time, amount of time spent asleep (min); WAIS‐III digit span backwards, digit span backwards from the Wechsler Adult Intelligence Scale‐Third Edition, where higher scores indicate better working memory; WASO, wake after sleep onset, amount of time spent awake after falling asleep (min).

^a^ Sleep discrepancy was calculated using difference scores, i.e. sleep diary – actigraphy. Note, WASO discrepancy scores were reversed as higher WASO indicates poorer sleep, while higher TST and SE reflect better sleep. Negative values for TST, WASO and SE discrepancy reflect a negative sleep discrepancy, whilst positive values reflect a positive sleep discrepancy. Mean values (≥ 5 nights) were used for all sleep parameters.

## 3 MATERIALS

### 3.1 Sleep assessment

*Self‐reported sleep* was measured with the Consensus Sleep Diary (Carney et al., 2012), which assesses sleep patterns over seven consecutive nights for: TST (min), WASO (min) and SE (%). TST was calculated by subtracting the sum of WASO (“In total, how long did these awakenings last?”) and SOL (“How long did it take you to fall asleep?”) from the sleep period (“What time did you try to go to sleep?” – “What time was your final awakening?”). SE was calculated by dividing TST by TIB (“What time did you get into bed?” – “What time did you get out of bed for the day?”).

*Objective sleep* was measured with actigraphy (Tri‐axial wGT3X‐BT activity monitor, Actigraph LLC, FL, USA). Actigraphy data were scored using ActiLife software (version 6.13.4) and Cole–Kripke algorithm (Cole et al., 1992), across 24‐h periods and 60‐s epochs. Bed/rise times were defined according to sleep diary estimates (“What time did you try to go to sleep?” and “What time was your final awakening?”). Data were visually inspected for missing or unusual values. If sleep diary bed/rise times were missing, or actigraphy data differed from sleep diary estimates of the sleep period by > 60 min, visual manual scoring was conducted in accordance with the Society of Behavioural Sleep Medicine guidelines (Ancoli‐Israel et al., 2015). Nights with non‐wear time within 5 min of bed/rise time or < 300 min of sleep opportunity were excluded from analyses.

All sleep parameters were continuous variables, with higher WASO, and lower TST and SE, indicating worse self‐reported and objective sleep. Mean values (≥ 5 nights) were used.

### 3.2 Cognitive assessment

*Delayed memory* was measured using the Delayed Memory Index from the Repeatable Battery for the Assessment of Neuropsychological Status (RBANS; Randolph et al., 1998), which includes List Recall, List Recognition, Story Recall, and Figure Recall subtests.

*Attention* was measured using the Attention Index from the RBANS (Randolph et al., 1998), consisting of Digit Span and Coding subtests. RBANS Index scores have a mean of 100 (SD = 15), and higher scores indicate greater ability.

*Executive function* was measured using a composite score derived from measures selected based on well‐established models of executive function (Fisk & Sharp, 2004; Friedman & Miyake, 2017). Specifically, working memory/updating, set‐shifting, response inhibition and generativity were assessed using Digit Span Backwards from the Wechsler Adult Intelligence Scale‐Third Edition (Wechsler, 1997), Trail Making Test – Part B (TMT‐B; Reitan, 1958), Flanker task from NIH‐EXAMINER (Kramer et al., 2014), and Letter Fluency (F only) from the Controlled Oral Word Association Test (Benton et al., 1983), respectively. Sample‐based *z*‐scores were calculated for each measure, then averaged to produce an overall measure of executive function, with higher scores (i.e. more positive *z*‐scores) indicating better performance. Given that less time to complete TMT‐B (seconds) reflects better performance, scores were reversed, such that higher scores for all executive function measures indicate better performance.

### 3.3 Other variables

*Insomnia severity* was assessed using the Insomnia Severity Index (ISI; Bastien et al., 2001), which evaluates insomnia symptoms over the past 2 weeks. Higher scores (0–28) indicate greater insomnia severity. *Depressive symptoms* were assessed using the Patient Health Questionnaire‐9 (PHQ‐9; Kroenke et al., 2001), which evaluates depression severity over the past 2 weeks. We omitted Item 3 (“trouble falling asleep or sleeping too much”) in our analyses, to separate the effects of sleep from depression; hereafter referred to as the PHQ‐8. Higher scores (0–24) indicate greater depressive symptomatology. *Obstructive sleep apnea (OSA) risk* was assessed using the Berlin Questionnaire (Netzer et al., 1999), which includes three categories (snoring, excessive daytime sleepiness, and high blood pressure/obesity) and classifies individuals as “high” or “low” OSA risk (coded as 1 and 0). All measures were selected due to their wide use and adequate psychometric properties (Ancoli‐Israel et al., 2003; Bastien et al., 2001; Carney et al., 2012; Kroenke et al., 2001; Netzer et al., 1999; Strauss et al., 2006).

### 3.4 Procedure

After providing written informed consent, participants were given sleep equipment (i.e. sleep diary and actigraph) and questionnaires to complete/wear across seven consecutive nights. Participants returned to the research lab to complete neuropsychological testing and were reimbursed for their time.

### 3.5 Statistical analyses

Cluster analysis was performed to characterise sleep discrepancy (using self‐reported sleep, objective sleep, and the difference score). To evaluate the relationship between sleep discrepancy and cognition, logistic regressions were conducted (with cognition predicting sleep discrepancy cluster membership), described in more detail below.

Statistical analyses were conducted using the R programming language (version 4.3.1; R Core Team, 2023). Missingness was approximately 2.77%; data were deemed to be missing at random based on visual inspection of an upset plot. Multiple imputation was performed using the multiple imputation by chained equations method (van Buuren & Groothuis‐Oudshoorn, 2011). All imputed values were deemed plausible, with no apparent bias/skew, based on visual inspection of data before/after imputations. Before conducting analyses, univariate outliers for continuous variables were identified based on a cut‐off of greater/less than 3 SD from the mean (*n* = 1 value for RBANS delayed memory index, *n* = 2 for executive function composite, *n* = 5 for PHQ‐8, *n* = 1 for education), and winsorized, while multivariate outliers (*n* = 5 participants for TST, *n* = 6 for WASO, *n* = 6 for SE) were determined using Mahalanobis distances using diary and actigraphy variables (*p* < 0.001) and removed. Other logistic regression assumptions were met.

#### 3.5.1 Sleep discrepancy calculation

To calculate sleep discrepancy, a difference score (i.e. sleep diary – actigraphy) was used. Given that lower WASO indicates better sleep quality, WASO difference scores were reflected such that negative values indicated a negative discrepancy. SOL and NWAK were omitted from further analyses: all participants had a negative SOL discrepancy, which may reflect a tendency for actigraphy to overestimate sleep in older adults (Martin & Hakim, 2011), and all participants had a positive NWAK discrepancy, which may reflect an inability for individuals to recall awakenings less than a certain duration (Winser et al., 2013).

#### 3.5.2 Cluster analyses

Agglomerative hierarchical clustering using a Euclidean distance and complete linkage method was performed using sleep diary, actigraphy and sleep discrepancy variables for TST, WASO and SE. Three scree plots were generated, and dendrograms were visually examined (i.e. elbow method) to determine the optimal number of clusters for each sleep parameter. This was cross‐referenced with a “majority vote” approach within the NbClust package/function (Charrad et al., 2014), which provides a frequency count for optimal clustering based on 30 different indices. Following this, the optimal number of clusters for each sleep parameter was fed into a tree‐cutting function that cut the dendrogram at the stipulated number of clusters and returned a vector assigning each participant to a cluster.

#### 3.5.3 Logistic regression

A two‐cluster solution was found for TST and SE, while a three‐cluster solution was observed for WASO. Therefore, binary logistic regressions (glm function, stats package; R Core Team, 2023) were utilised for TST and SE clusters, and multinomial logistic regression (multinom function, nnet package; Venables & Ripley, 2002) was employed for WASO, with the “no sleep discrepancy and good sleep quality” group as a reference. The unique predictive ability of cognition (i.e. memory, attention and executive function) on sleep discrepancy cluster membership was evaluated, controlling for OSA risk, age, sex and education (Model 1). Two subsequent models were performed, controlling for insomnia severity (Model 2) and depressive symptoms (Model 3), respectively. To address our exploratory aims, the following models were performed for each sleep parameter:

1. Sleep discrepancy ~ memory + attention + executive function + covariates
2. Sleep discrepancy ~ memory + attention + executive function + insomnia severity + covariates
3. Sleep discrepancy ~ memory + attention + executive function + depressive symptoms + covariates

#### 3.5.4 Post‐hoc exploratory analyses

After completing our planned analyses, questions regarding the role of objective sleep in the sleep discrepancy–cognition relationship were identified. Given the well‐established link between objective sleep and cognition in previous research (Rasch & Born, 2013), and that sleep discrepancy clusters were formed using a combination of self‐reported sleep, objective sleep and the difference score, we sought to explore whether the relationship between sleep discrepancy and cognition would remain when controlling for objective sleep (Model 4).

4 Sleep discrepancy ~ memory + attention + executive function + objective sleep + covariates

Given that the current study is exploratory, adjustments for multiple comparisons were not performed (Althouse, 2016).

## 4 RESULTS

### 4.1 Cluster analyses and exploration

**FIGURE 1:**
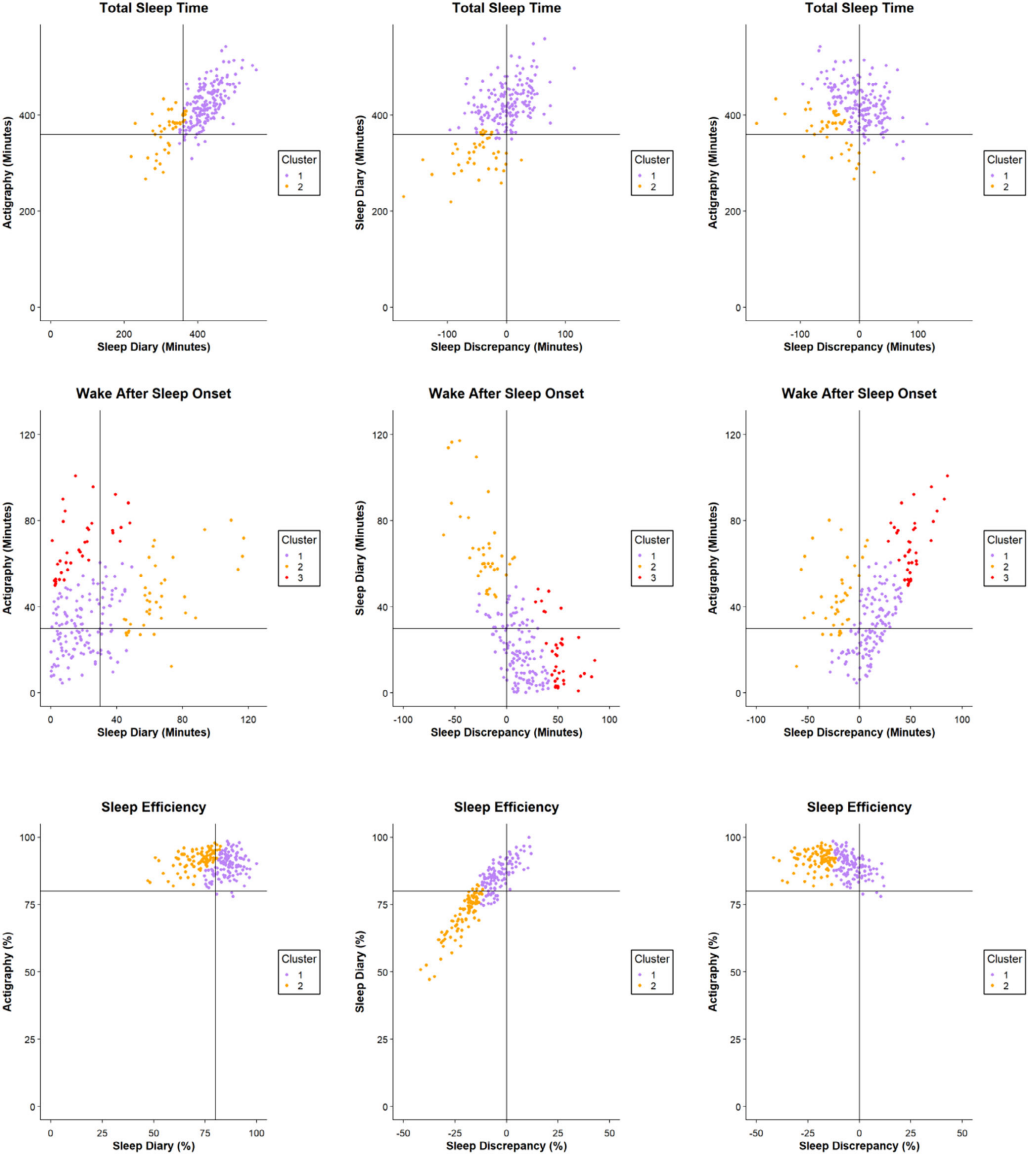
Scatter plots of self‐reported sleep (i.e. sleep diary), objective sleep (i.e. actigraphy) and sleep discrepancy characteristics. Mean values (≥ 5 nights) were used for all sleep parameters. SE, sleep efficiency (ratio of total sleep time [TST] to time in bed [TIB], %); TST, total sleep time (amount of time spent asleep, min); WASO, wake after sleep onset (amount of time spent awake after falling asleep, min). The colouring scheme depicts a two‐cluster solution for TST, where: Cluster 1 (purple) = “TST 1: No sleep discrepancy and longer sleep duration”; Cluster 2 (orange) = “TST 2: Negative sleep discrepancy and shorter sleep duration”. For WASO, there is a three‐cluster solution, where Cluster 1 (purple) = “WASO 1: No sleep discrepancy and good sleep quality”; Cluster 2 (orange) = “WASO 2: Negative sleep discrepancy and poor sleep quality”; Cluster 3 (red) = “WASO 1: Positive sleep discrepancy and poor sleep quality”. For SE, there is a two‐cluster solution, where: Cluster 1 (purple) = “SE 1: No sleep discrepancy and good self‐reported sleep quality”; Cluster 2 (orange) = “SE 2: Negative sleep discrepancy and poor self‐reported sleep quality”. Sleep discrepancy was calculated using difference score (i.e. sleep discrepancy = sleep diary – actigraphy). Sleep discrepancy was calculated using difference scores (i.e. sleep diary – actigraphy). Note, WASO scores were reversed as higher WASO indicates poorer sleep, while higher TST and SE reflect better sleep. Negative values for TST, WASO and SE discrepancy reflect a negative sleep discrepancy, where self‐reported sleep is worse than objective sleep, whilst positive values reflect a positive sleep discrepancy, where self‐reported sleep is better than objective sleep. Solid black horizontal and vertical lines correspond to cut‐offs consistent with National Sleep Foundation guidelines for adequate sleep.

For all sleep parameters, clusters were labelled according to statistical differences observed in the contingency tables (Tables S1.1–S3.3), in conjunction with visual inspection of the scatterplots (Figure 1) and descriptive statistics (Table 2).

**TABLE 2:** Self‐reported sleep, objective sleep and sleep discrepancy across TST, WASO and SE clusters.

	TST (*N* = 216)				WASO (*N* = 215)						SE (*N* = 215)			
	Cluster 1		Cluster 2		Cluster 1		Cluster 2		Cluster 3		Cluster 1		Cluster 2	
	(*n* = 174)		(*n* = 42)		(*n* = 140)		(*n* = 39)		(*n* = 36)		(*n* = 122)		(*n* = 93)	
	Mean (SD)	Range	Mean (SD)	Range	Mean (SD)	Range	Mean (SD)	Range	Mean (SD)	Range	Mean (SD)	Range	Mean (SD)	Range
Sleep diary	425.87 (39.76)**	350.00–558.57	317.27 (36.61)**	219.14–368.14	19.44 (12.82)**	0.21–49.29	67.27 (19.65)**	44.54–117.14	17.70 (14.24)**	0.86–48.14	86.11 (5.27)**	74.65–100.00	71.09 (7.80)**	47.21–82.35
Actigraphy	424.28 (41.90)**	309.29–542.51	366.18 (41.43)**	267.14–434.00	31.22 (13.37)**	4.57–60.43	45.01 (15.91)**	12.28–80.23	69.08 (13.79)**	50.00–100.71	89.55 (4.33)**	78.00–100.00	91.71 (3.47)**	81.89–97.89
Sleep	1.53 (34.18)**	−95.74 – 115.29	−50.25 (38.69)**	−174.50 – 25.14	11.78 (14.61)**	−28.14 – 40.57	−22.26 (16.48)**	−61.00 – 7.89	51.38 (13.17)**	28.29–85.71	−3.43 (5.75)**	−12.75 – 12.03	−20.64 (6.68)**	−41.64 – −11.61
Discrepancy^a^														

*Note*: For TST, Cluster 1 = “TST 1: no sleep discrepancy and longer sleep duration”; Cluster 2 = “TST 2: negative sleep discrepancy and shorter sleep duration”. For WASO, Cluster 1 = “WASO 1: no sleep discrepancy and good sleep quality”; Cluster 2 = “WASO 2: negative sleep discrepancy and poor sleep quality”; Cluster 3 = “WASO 3: positive sleep discrepancy and poor sleep quality”. For SE, Cluster 1 = “SE 1: negative sleep discrepancy and poor self‐reported sleep quality”; Cluster 2 = “SE: no sleep discrepancy and good self‐reported sleep quality”. Mean values (≥ 5 nights) were used for all sleep parameters.

Abbreviations: SE, sleep efficiency, ratio of total sleep time to time in bed (%); TST, total sleep time, amount of time spent asleep (min); WASO, wake after sleep onset, amount of time spent awake after falling asleep (min).

^a^ Sleep discrepancy was calculated using difference scores (i.e. sleep diary – actigraphy). Note, WASO discrepancy scores were reversed as higher WASO indicates poorer sleep, while higher TST and SE reflect better sleep. Negative values for TST, WASO and SE discrepancy reflect a negative sleep discrepancy, where self‐reported sleep is worse than objective sleep, whilst positive values reflect a positive sleep discrepancy, where self‐reported sleep is better than objective sleep. Independent‐samples *t*‐tests and analysis of variance (ANOVA) were performed to compare continuous variable means across clusters.

** *p* < 0.01.

#### 4.1.1 Total sleep time

A two‐cluster solution for TST is displayed in Figure 1. Descriptive statistics for self‐reported TST, objective TST and TST discrepancy, by cluster, are shown in Table 2. Additionally, 2 × 2 contingency tables, where frequency counts for each cluster were split across known cut‐offs for self‐reported/objective TST (i.e. 6 hr; Hirshkowitz et al., 2015), and zero for TST discrepancy, are presented in Tables S1.1–S3.3. Fisher's exact tests were statistically significant, confirming differences across clusters in self‐reported TST, objective TST and TST discrepancy (all *p* < 0.001). Relative to Cluster 1, Cluster 2 was characterised by shorter self‐reported and objective sleep duration, and greater negative discrepancy.

Cluster 1 (purple) was labelled “TST 1: no sleep discrepancy and longer sleep duration”, given that its members had a relatively low sleep discrepancy mean (1.58 min). Additionally, this cluster's self‐reported and objective sleep means were both greater than those of Cluster 2. Further, participants grouped towards the 0‐min cut‐off indicating no discrepancy, and most were above the 360‐min cut‐off indicating longer self‐reported/objective sleep duration (Figure 1). Cluster 2 (orange) was labelled “TST 2: negative sleep discrepancy and shorter sleep duration”, as the direction of sleep discrepancy was almost exclusively negative, bar one participant, and this cluster had the lowest self‐reported and objective sleep mean.

#### 4.1.2 Wake after sleep onset

A three‐cluster solution was found for WASO (Figure 1). Descriptive statistics for each cluster are shown in Table 2. Further, 3 × 2 contingency tables, where frequency counts for each cluster were split across known cut‐offs for self‐reported/objective WASO (i.e. 30 min; Ohayon et al., 2017), and zero for WASO discrepancy, are reported in Tables S2.1‐S2.3. Fisher's exact tests were statistically significant, revealing differences across clusters in self‐reported WASO, objective WASO and WASO discrepancy (all *p* < 0.001). Fisher's exact tests showed significant differences across pairwise cluster comparisons for all sleep variables (i.e. self‐reported WASO, objective WASO and WASO discrepancy), with one exception: Clusters 1 and 3 did not differ in self‐reported sleep (*p* = 0.66). Relative to Cluster 1, Cluster 3 was characterised by poorer objective sleep quality and greater positive discrepancy, while Cluster 2 was characterised by poorer self‐reported and objective sleep quality and greater negative discrepancy. Additionally, relative to Cluster 2, Cluster 3 was characterised by better self‐reported sleep but worse objective sleep (i.e. positive discrepancy).

Cluster 1 (purple) was labelled “WASO 1: no sleep discrepancy and good sleep quality”, given that its members exhibited the lowest sleep discrepancy mean of all three clusters. Participants tended to group towards the 0‐min cut‐off indicating no discrepancy (Figure 1). Cluster 2 (orange) was labelled “WASO 2: negative sleep discrepancy and poor sleep quality” given that most members (92%) showed a negative discrepancy and that it is the only WASO cluster characterised by a negative discrepancy mean (−22.26 min). Additionally, all members' self‐reported WASO, and most members' objective WASO, were above the 30‐min cut‐off, indicating poor sleep quality. Cluster 3 (red) was labelled “WASO 3: positive sleep discrepancy and poor sleep quality” as all participants demonstrated positive discrepancy, with this being the only WASO cluster with a positive discrepancy mean (51.38 min). Additionally, all participants were well above the 30‐min cut‐off for objective WASO, indicating poor sleep quality (Figure 1).

#### 4.1.3 Sleep efficiency

A two‐cluster solution for SE is displayed in Figure 1. Descriptive statistics for each cluster are shown in Table 2. Further, 2 × 2 contingency tables can be seen in Tables S3.1‐S3.3, where frequency counts for each cluster were split across known cut‐offs for self‐reported/objective SE (i.e. 80%; Ohayon et al., 2017), and zero for SE discrepancy. Fisher's exact tests were statistically significant for two of three variables, confirming differences across clusters in self‐reported SE and SE discrepancy (both *p* < 0.001), but not objective SE (*p* = 0.26); therefore, only differences in self‐reported SE and SE discrepancy were considered. Relative to Cluster 1, Cluster 2 was characterised by poorer self‐reported sleep quality and greater negative discrepancy.

Cluster 1 was labelled “SE 1: no sleep discrepancy and good self‐reported sleep quality”, given its relatively low sleep discrepancy mean (−3.43%) and most members (85%) having a self‐reported SE of > 80% indicating good sleep quality. Indeed, participants were grouped towards the 0‐min cut‐off indicating no discrepancy, and most were above the 80% cut‐off indicating good self‐reported sleep quality (Figure 1). In contrast, Cluster 2 was labelled “SE 2: negative sleep discrepancy and poor self‐reported sleep quality” due to all members demonstrating a negative discrepancy (mean = −20.64%). Most members (95%) had a self‐reported SE of < 80%, indicating poor sleep quality.

Sample characteristics across clusters are shown in Table 3. To compare continuous variable means across clusters, independent samples *t*‐tests (for TST and SE) and one‐way between‐subjects analysis of variance (for WASO) were performed. Fisher's exact tests were performed to compare proportions of sex (male/female) and OSA risk (high/low) across clusters.

**TABLE 3:** Sample characteristics across TST, WASO and SE clusters.

Variable	TST (*N* = 216)				WASO (*N* = 215)						SE (*N* = 215)			
	Cluster 1 (*n* = 174)		Cluster 2 (*n* = 42)		Cluster 1 (*n* = 140)		Cluster 2 (*n* = 39)		Cluster 3 (*n* = 36)		Cluster 1 (*n* = 122)		Cluster 2 (*n* = 93)	
	Mean (SD) or *N* (%)	Range	Mean (SD) or *N* (%)	Range	Mean (SD) or *N* (%)	Range	Mean (SD) or *N* (%)	Range	Mean (SD) or *N* (%)	Range	Mean (SD) or *N* (%)	Range	Mean (SD) or *N* (%)	Range
Age, years	71.62 (7.59)	55–93	72.74 (6.79)	56–88	72.36 (7.31)*	55–92	73.49 (7.82)*	55–93	68.06 (6.41)*	55–83	70.81 (6.94)*	55–93	73.18 (7.93)*	55–90
Sex, *N* = female (%)	106 (60.92)	–	30 (71.43)	–	92 (65.71)	–	23 (58.97)	–	22 (61.11)	–	74 (60.66)	–	63 (67.74)	–
Education, years	13.89 (3.16)	3–20	13.70 (3.35)	7–20	14.00 (3.07)	3–20	13.39 (3.94)	7–20	13.64 (2.77)	8–19	13.97 (3.08)	3–20	13.74 (3.29)	6–20
GAD‐7 total score	1.58 (2.32)	0–14	2.67 (3.73)	0–16	1.49 (2.26)	0–14	2.41 (3.45)	0–16	2.11 (3.09)	0–14	1.72 (2.40)	0–14	1.88 (3.02)	0–16
PHQ‐9 total score	2.37 (2.89)*	0–13	3.36 (2.52)*	0–11	1.96 (2.10)**	0–13	3.51 (2.92)**	0–12	3.81 (4.24)**	0–13	2.52 (3.18)	0–13	2.61 (2.36)	0–11
BMI, kg m^−2^	26.29 (3.87)	18.20–49.95	26.20 (5.49)	18.37–48.37	25.91 (4.15)*	18.20–48.37	25.56 (3.32)*	19.06–33.13	28.15 (4.97)*	21.43–49.95	26.62 (4.01)	18.92–49.95	25.83 (4.51)	18.20–48.37
ISI total score	6.51 (6.18)**	0–26	12.10 (5.66)**	1–25	6.73 (6.05)	0–25	10.69 (7.33)	1–25	7.08 (6.20)	0–26	6.15 (5.79)**	0–26	9.27 (6.88)**	0–25
Berlin risk score, *N* = low OSA risk (%)	118 (67.82)	–	28 (66.67)	–	101 (67.33)*	–	27 (69.23)*	–	18 (50.00)*	–	79 (64.75)	–	67 (72.04)	–
RBANS Delayed Memory index	102.20 (11.99)	64–126	100.00 (13.39)	60–121	103.44 (11.88)*	64–126	98.85 (12.63)*	71–121	98.56 (11.95)*	71–126	103.37 (12.09)*	64–126	100.09 (12.04)*	68–126
RBANS Attention index	106.45 (14.75)	72–142	109.78 (13.82)	82–142	107.85 (14.52)	72–142	106.91 (15.75)	82–142	104.14 (13.78)	75–135	107.89 (14.34)	79–142	106.50 (14.68)	72–142
EF composite	0.04 (1.02)	−3.21 – 2.63	−0.10 (0.90)	−2.06 – 2.65	0.02 (0.95)	−3.21 – 2.59	0.01 (1.19)	−2.16 – 2.65	−0.04 (0.99)	−2.68 – 1.61	0.05 (1.09)	−3.21 – 2.65	−0.03 (0.88)	−2.06 – 2.27
MMSE total score	27.94 (1.52)	22–30	27.92 (1.81)	22–30	27.99 (1.45)	22–30	27.87 (1.65)	24–30	28.50 (1.90)	22–30	28.00 (1.57)	22–30	27.89 (1.57)	22–30

*Note*: For TST, Cluster 1 = “TST 1: no sleep discrepancy and longer sleep duration”; Cluster 2 = “TST 2: negative sleep discrepancy and shorter sleep duration”. For WASO, Cluster 1 = “WASO 1: no sleep discrepancy and good sleep quality”; Cluster 2 = “WASO 2: negative sleep discrepancy and poor sleep quality”; Cluster 3 = “WASO 3: positive sleep discrepancy and poor sleep quality”. For SE, Cluster 1 = “SE 1: negative sleep discrepancy and poor self‐reported sleep quality”; Cluster 2 = “SE 2: no sleep discrepancy and good self‐reported sleep quality”. Independent‐samples *t*‐tests and analysis of variance (ANOVA) were performed to compare continuous variable means across clusters, while Fisher's exact tests were performed to compare proportions for categorical variables.

Abbreviations: Berlin, Berlin Questionnaire, measure of OSA risk; BMI, body mass index; EF composite, executive function composite comprised of tests measuring working memory, set‐shifting, response inhibition and generativity; GAD‐7, Generalised Anxiety Disorder, where higher scores indicate greater anxiety symptoms; ISI, Insomnia Severity Index, where higher scores indicate greater insomnia severity; MMSE, Mini Mental State Examination, where higher scores indicate greater global cognitive function; OSA, obstructive sleep apnea; PHQ‐9, Patient Health Questionnaire, where higher scores indicate greater depressive symptoms; RBANS, Repeatable Battery for the Assessment of Neuropsychological Status, where higher Attention and Delayed Memory Index scores suggest greater attention and delayed memory ability, respectively; SE, sleep efficiency, ratio of total sleep time to time in bed (%); TST, total sleep time, amount of time spent asleep (min); WASO, wake after sleep onset, amount of time spent awake after falling asleep (min).

* *p* < 0.05.

** *p* < 0.01.

### 4.2 Predicting sleep discrepancy cluster membership with cognition

A summary of regression output for the TST, WASO and SE models, including beta‐weights, odds ratios and *p*‐values, is shown in Tables 4, 5, 6. This includes relationships between covariates (e.g. insomnia severity and depressive symptoms) with sleep discrepancy clusters across TST, WASO and SE models.

**TABLE 4:** Binary logistic regression analyses exploring cognitive function, sleep characteristics and demographics as a predictor of TST cluster membership.

Variable	Model 1				Model 2				Model 3				Model 4			
	*β*	SE	OR [95% ECI]	*p*	*β*	SE	OR [95% ECI]	*p*	*β*	SE	OR [95% ECI]	*p*	*β*	SE	OR [95% ECI]	*p*
Age	0.02	0.03	1.03 [0.98, 1.08]	0.33	0.03	0.03	1.03 [0.98, 1.09]	0.24	0.03	0.03	1.03 [0.98, 1.09]	0.23	0.04	0.03	1.04 [0.98, 1.11]	0.21
Sex	0.62	0.41	1.86 [0.86, 4.29]	0.13	0.63	0.45	1.86 [0.79, 4.68]	0.17	0.60	0.41	1.82 [0.83, 4.22]	0.14	0.65	0.50	1.91 [0.74, 5.33]	0.19
Education	−0.01	0.06	0.99 [0.88, 1.11]	0.91	−0.01	0.06	0.99 [0.87, 1.12]	0.84	−0.01	0.06	0.99 [0.88, 1.11]	0.87	−0.04	0.07	0.96 [0.83, 1.10]	0.55
Berlin risk score	0.16	0.38	1.18 [0.55, 2.45]	0.67	−0.71	0.47	0.49 [0.19, 1.18]	0.13	0.07	0.39	1.08 [0.49, 2.28]	0.85	0.20	0.47	1.22 [0.48, 3.03]	0.66
RBANS delayed memory index	−0.26	0.19	0.77 [0.53, 1.12]	0.17	−0.18	0.20	0.84 [0.56, 1.26]	0.39	−0.22	0.19	0.80 [0.55, 1.16]	0.24	−0.15	0.24	0.86 [0.54, 1.38]	0.53
RBANS attention index	0.30	0.21	1.35 [0.90, 2.04]	0.15	0.40	0.22	1.49 [0.97, 2.32]	0.07	0.32	0.21	1.37 [0.92, 2.08]	0.13	0.51	0.27	1.66 [0.99, 2.85]	0.06
EF composite	−0.08	0.23	0.93 [0.598, 1.48]	0.75	−0.21	0.26	0.81 [0.48, 1.32]	0.39	−0.06	0.24	0.94 [0.59, 1.50]	0.79	−0.11	0.30	0.89 [0.50, 1.61]	0.70
ISI total score	–	–	–	–	0.15	0.03	1.17 [1.10, 1.25]	< 0.001^**^	–	–	–	–	–	–	–	–
PHQ‐8 total score	–	–	–	–	–	–	–	–	0.10	0.08	1.10 [0.94, 1.28]	0.21	–	–	–	–
Actigraphy	–	–	–	–	–	–	–	–	–	–	–	–	−0.04	0.01	0.96 [0.95, 0.97]	< 0.001^**^

*Note*: *N* = 216; Sleep discrepancy was calculated using difference scores (i.e. sleep diary – actigraphy). Negative values for TST discrepancy reflect a negative sleep discrepancy, where self‐reported sleep is worse than objective sleep, whilst positive values reflect a positive sleep discrepancy, where self‐reported sleep is better than objective sleep. For sex (male/female) and the Berlin Questionnaire (high/low), values are coded such that females = 1 and individuals with high risk of OSA = 1. Age and education are in years. For TST, there is a two‐cluster solution, where: Cluster 1 = “TST 1: no sleep discrepancy and longer sleep duration”; Cluster 2 = “TST 2: negative sleep discrepancy and shorter sleep duration”. Model 1 = memory, attention and executive function predicting TST discrepancy cluster membership, controlling for age, sex, education and OSA risk. Model 2 = memory, attention and executive function predicting TST discrepancy cluster membership, controlling for age, sex, education, OSA risk and insomnia severity. Model 3 = memory, attention and executive function predicting TST discrepancy cluster membership, controlling for age, sex, education, OSA risk and depressive symptoms. Model 4 = memory, attention and executive function predicting TST discrepancy cluster membership, controlling for age, sex, education, OSA risk and objective sleep.

Abbreviations: Actigraphy, measure of objective sleep; *β*, unstandardised beta‐weight (except for analyses involving the RBANS attention index, RBANS delayed memory index and EF composite, which were based on *z*‐scores); Berlin, Berlin Questionnaire, measure of OSA risk; ECI, exponentiated confidence interval; EF composite, executive function composite comprised of working memory, set‐shifting, response inhibition and generativity, with higher scores suggesting greater EF; ISI total score, Insomnia Severity Index total score, where higher scores indicate greater insomnia severity; OR, odds ratio; PHQ‐8 total score, Patient Health Questionnaire minus the sleep item, where higher scores indicate greater depressive symptoms; RBANS, Repeatable Battery for the Assessment of Neuropsychological Status, where higher Attention and Delayed Memory Index scores indicate greater attention and delayed memory ability, respectively; SE, standard error.

** *p* < 0.01.

**TABLE 5:** Multinomial logistic regression analyses exploring cognitive function, sleep characteristics and demographics as predictors of WASO cluster membership.

Variable	Negative discrepancy				Positive discrepancy			
	*β*	SE	OR [95% ECI]	*p*	*β*	SE	OR [95% ECI]	*p*
Model 1								
Age	0.03	0.03	1.03 [0.98, 1.09]	0.27	−0.11	0.03	0.90 [0.84, 0.96]	0.001^**^
Sex	−0.19	0.40	0.83 [0.38, 1.81]	0.63	−0.09	0.44	0.92 [0.39, 2.15]	0.84
Education	−0.07	0.07	0.93 [0.83, 1.05]	0.26	−0.001	0.07	1.00 [0.87, 1.15]	0.99
Berlin risk score	0.07	0.41	1.07 [0.48, 2.40]	0.86	0.80	0.41	2.23 [1.00, 4.96]	0.05
RBANS delayed memory index	−0.44	0.20	0.65 [0.44, 0.96]	0.03*	−0.34	0.21	0.71 [0.47, 1.08]	0.11
RBANS attention index	−0.11	0.21	0.89 [0.59, 1.36]	0.60	−0.23	0.25	0.80 [0.49, 1.31]	0.37
EF composite	0.40	0.25	1.49 [0.91, 2.44]	0.12	−0.18	0.26	0.83 [0.50, 1.38]	0.48
Model 2								
Age	0.04	0.03	1.04 [0.98, 1.10]	0.18	−0.11	0.03	0.90 [0.84, 0.96]	0.001^**^
Sex	−0.27	0.42	0.76 [0.34, 1.72]	0.51	−0.03	0.44	0.97 [0.41, 2.32]	0.95
Education	−0.07	0.06	0.93 [0.82, 1.05]	0.24	−0.01	0.07	0.99 [0.86, 1.15]	0.94
Berlin risk score	−0.48	0.47	0.62 [0.25, 1.55]	0.31	0.86	0.43	2.36 [1.02, 5.44]	0.04*
RBANS delayed memory index	−0.39	0.21	0.67 [0.45, 1.02]	0.06	−0.34	0.21	0.71 [0.47, 1.08]	0.11
RBANS attention index	−0.06	0.22	0.94 [0.61, 1.44]	0.78	−0.25	0.25	0.78 [0.48, 1.28]	0.33
EF composite	0.35	0.26	1.42 [0.85, 2.36]	0.18	−0.15	0.26	0.86 [0.52, 1.43]	0.57
ISI total score	0.10	0.03	1.10 [1.04, 1.17]	0.001^**^	−0.02	0.03	0.98 [0.91, 1.05]	0.52
Model 3								
Age	0.05	0.03	1.05 [0.99, 1.11]	0.11	−0.09	0.03	0.91 [0.85, 0.97]	0.01^**^
Sex	−0.30	0.41	0.74 [0.33, 1.66]	0.47	−0.19	0.44	0.83 [0.35, 1.97]	0.67
Education	−0.08	0.06	0.92 [0.81, 1.04]	0.19	−0.004	0.07	1.00 [0.87, 1.15]	0.99
Berlin risk score	−0.07	0.42	0.94 [0.41, 2.14]	0.88	0.68	0.42	1.97 [0.87, 4.47]	0.10
RBANS delayed memory index	−0.35	0.21	0.70 [0.47, 1.05]	0.09	−0.30	0.21	0.74 [0.48, 1.12]	0.16
RBANS attention index	−0.09	0.22	0.91 [0.59, 1.40]	0.67	−0.20	0.25	0.82 [0.50, 1.35]	0.43
EF composite	0.46	0.26	1.58 [0.95, 2.63]	0.08	−0.11	0.27	0.90 [0.53, 1.52]	0.69
PHQ‐8 total score	0.23	0.09	1.26 [1.07, 1.49]	0.01^**^	0.19	0.08	1.20 [1.02, 1.42]	0.03*
Model 4								
Age	0.03	0.03	1.03 [0.97, 1.09]	0.38	−0.12	0.06	0.88 [0.79, 0.99]	0.03*
Sex	−0.28	0.45	0.75 [0.31, 1.83]	0.53	0.17	0.72	1.19 [0.29, 4.87]	0.81
Education	−0.13	0.07	0.87 [0.76, 1.00]	0.05	−0.05	0.12	0.95 [0.75, 1.21]	0.68
Berlin risk score	−0.27	0.45	0.76 [0.31, 1.85]	0.55	0.09	0.68	1.10 [0.29, 4.19]	0.89
RBANS delayed memory index	−0.58	0.23	0.56 [0.36, 0.88]	0.01*	−0.62	0.38	0.54 [0.25, 1.14]	0.11
RBANS attention index	−0.005	0.25	1.00 [0.61, 1.61]	0.98	−0.06	0.40	0.94 [0.43, 2.05]	0.88
EF composite	0.47	0.30	1.60 [0.89, 2.86]	0.11	−0.33	0.45	0.72 [0.30, 1.76]	0.47
Actigraphy	0.08	0.02	1.22 [1.05, 1.12]	< 0.001^**^	0.20	0.03	1.22 [1.15, 1.29]	< 0.001^**^

*Note*: Sleep discrepancy was calculated using difference scores (i.e. sleep diary – actigraphy). WASO discrepancy scores were reversed, given that higher WASO indicates worse sleep quality. Negative values for WASO discrepancy reflect a negative sleep discrepancy, where self‐reported sleep is worse than objective sleep, whilst positive values reflect a positive sleep discrepancy, where self‐reported sleep is better than objective sleep. All analyses were compared against the “no sleep discrepancy and good sleep quality” cluster as a reference group. For sex (male/female) and the Berlin Questionnaire (high/low), values are coded such that females = 1 and individuals with high risk of OSA = 1. Age and education are in years. For WASO, there is a three‐cluster solution, where: Cluster 1 = “WASO 1: no sleep discrepancy and good sleep quality”; Cluster 2 = “WASO 2: negative sleep discrepancy and poor sleep quality”; Cluster 3 = “WASO 3: positive sleep discrepancy and poor sleep quality”. Model 1 = memory, attention and executive function predicting WASO discrepancy cluster membership, controlling for age, sex, education and OSA risk. Model 2 = memory, attention and executive function predicting WASO discrepancy cluster membership, controlling for age, sex, education, OSA risk and insomnia severity. Model 3 = memory, attention and executive function predicting WASO discrepancy cluster membership, controlling for age, sex, education, OSA risk and depressive symptoms. Model 4 = memory, attention and executive function predicting WASO discrepancy cluster membership, controlling for age, sex, education, OSA risk and objective sleep.

Abbreviations: Actigraphy, measure of objective sleep; *β*, unstandardised beta‐weight (except for analyses involving the RBANS attention index, RBANS delayed memory index and EF composite, which were based on *z*‐scores); Berlin, Berlin Questionnaire, measure of OSA risk; ECI, exponentiated confidence interval; EF composite, executive function composite comprised of working memory, set‐shifting, response inhibition and generativity, with higher scores suggesting greater EF; ISI total score, Insomnia Severity Index total score, where higher scores indicate greater insomnia severity; OR, odds ratio; PHQ‐8 total score, Patient Health Questionnaire minus the sleep item, where higher scores indicate greater depressive symptoms; RBANS, Repeatable Battery for the Assessment of Neuropsychological Status, where higher Attention and Delayed Memory Index scores indicate greater attention and delayed memory ability, respectively; SE, standard error.

* *p* < 0.05.

** *p* < 0.01.

**TABLE 6:** Binary logistic regression analyses exploring cognitive function, sleep characteristics and demographics as predictors of SE cluster membership.

Variable	Model 1				Model 2				Model 3				Model 4			
	*β*	SE	OR [95% ECI]	*P*	*Β*	SE	OR [95% ECI]	*p*	*β*	SE	OR [95% ECI]	*p*	*β*	SE	OR [95% ECI]	*p*
Age	0.06	0.02	1.06 [1.02, 1.11]	0.01^**^	0.06	0.02	1.07 [1.02, 1.11]	0.01^**^	0.05	0.02	1.06 [1.01, 1.10]	0.01*	0.05	0.02	1.06 [1.01, 1.10]	0.01*
Sex	0.50	0.32	1.66 [0.89, 3.12]	0.11	0.44	0.33	1.55 [0.81, 3.00]	0.19	0.52	0.32	1.68 [0.91, 3.18]	0.10	0.56	0.33	1.74 [0.92, 3.36]	0.09
Education	−0.01	0.05	0.99 [0.90, 1.09]	0.83	−0.01	0.05	0.99 [0.90, 1.09]	0.84	−0.01	0.05	0.99 [0.90, 1.09]	0.85	0.003	0.05	1.00 [0.91, 1.10]	0.94
Berlin risk score	−0.28	0.31	0.76 [0.41, 1.40]	0.38	−0.76	0.36	0.47 [0.23, 0.92]	0.03*	−0.23	0.32	0.79 [0.42, 1.48]	0.46	−0.07	0.33	0.93 [0.48, 1.78]	0.83
RBANS delayed memory index	−0.39	0.16	0.68 [0.49, 0.93]	0.02*	−0.35	0.17	0.70 [0.50, 0.97]	0.04*	−0.41	0.17	0.66 [0.47, 0.91]	0.01*	−0.42	0.17	0.65 [0.46, 0.90]	0.01*
RBANS attention index	−0.17	0.17	0.84 [0.60, 1.18]	0.32	−0.15	0.18	0.86 [0.61, 1.22]	0.40	−0.18	0.17	0.84 [0.59, 1.17]	0.29	−0.22	0.18	0.81 [0.57, 1.14]	0.22
EF composite	0.29	0.19	1.33 [0.92, 1.96]	0.14	0.23	0.20	1.26 [0.85, 1.88]	0.26	0.28	0.19	1.32 [0.91, 1.95]	0.15	0.31	0.20	1.37 [0.93, 2.04]	0.12
ISI total score	–	–	–	–	0.10	0.03	1.11 [1.05, 1.17]	< 0.001^**^	–	–	–	–	–	–	–	–
PHQ‐8 total score	–	–	–	–	–	–	–	–	−0.05	0.07	0.95 [0.82, 1.08]	0.42	–	–	–	–
Actigraphy	–	–	–	–	–	–	–	–	–	–	–	–	0.14	0.04	1.15 [1.07, 1.25]	< 0.001^**^

*Note*: *N* = 215; Sleep discrepancy was calculated using difference scores (i.e. sleep diary – actigraphy). Negative values for SE discrepancy reflect a negative sleep discrepancy, where self‐reported sleep is worse than objective sleep, whilst positive values reflect a positive sleep discrepancy, where self‐reported sleep is better than objective sleep. For sex (male/female) and the Berlin Questionnaire (high/low), values are coded such that females = 1 and individuals with high risk of OSA = 1. Age and education are in years. For SE, there is a two‐cluster solution, where: Cluster 1 = “SE 1: no sleep discrepancy and good sleep quality”; Cluster 2 = “SE 2: negative sleep discrepancy and poor sleep quality”. Model 1 = memory, attention and executive function predicting SE discrepancy cluster membership, controlling for age, sex, education and OSA risk. Model 2 = memory, attention and executive function predicting SE discrepancy cluster membership, controlling for age, sex, education, OSA risk and insomnia severity. Model 3 = memory, attention and executive function predicting SE discrepancy cluster membership, controlling for age, sex, education, OSA risk and depressive symptoms. Model 4 = memory, attention and executive function predicting SE discrepancy cluster membership, controlling for age, sex, education, OSA risk and objective sleep.

Abbreviations: Actigraphy, measure of objective sleep; *β*, unstandardised beta‐weight (except for analyses involving the RBANS attention index, RBANS delayed memory index and EF composite, which were based on *z*‐scores); Berlin, Berlin Questionnaire, measure of OSA risk; ECI, exponentiated confidence interval; EF composite, executive function composite comprised of working memory, set‐shifting, response inhibition and generativity, with higher scores suggesting greater EF; ISI total score, Insomnia Severity Index total score, where higher scores indicate greater insomnia severity; OR, odds ratio; PHQ‐8 total score, Patient Health Questionnaire minus the sleep item, where higher scores indicate greater depressive symptoms; RBANS, Repeatable Battery for the Assessment of Neuropsychological Status, where higher Attention and Delayed Memory Index scores indicate greater attention and delayed memory ability, respectively; SE, standard error.

* *p* < 0.05.

** *p* < 0.01.

#### 4.2.1 Total sleep time

No significant relationships were found between cognition and TST discrepancy cluster membership, both before and after controlling for insomnia severity and depressive symptoms, respectively (Models 1–3).

#### 4.2.2 Wake after sleep onset

Poorer delayed memory was associated with a greater likelihood of belonging to the “WASO 2: negative sleep discrepancy and poor sleep quality” versus “WASO 1: no sleep discrepancy and good sleep quality” group, with a medium effect size (Model 1). When controlling for insomnia severity and depressive symptoms, respectively, the relationship between delayed memory and sleep discrepancy cluster membership became non‐significant (Models 2 and 3). Meanwhile, there were no significant relationships between cognition and positive WASO discrepancy cluster membership, including when controlling for insomnia severity and depressive symptoms, respectively (Models 1–3).

#### 4.2.3 Sleep efficiency

Poorer delayed memory was associated with a greater likelihood of belonging to the “SE 2: negative sleep discrepancy and poor self‐reported sleep quality” versus “SE 1: no sleep discrepancy and good self‐reported sleep quality” group; insomnia severity and depressive symptoms did not affect this association, with medium effect sizes observed (Models 1–3).

#### 4.2.4 Post‐hoc exploratory analyses

When controlling for objective sleep, the same pattern of results was found across all sleep parameters (Tables 4, 5, 6, Model 4). There were significant associations between poorer delayed memory with both negative WASO discrepancy and negative SE discrepancy, with medium–large effect sizes. However, there was no relationship between cognition and positive WASO discrepancy, or negative TST discrepancy. Although the relationship between negative WASO discrepancy and poorer delayed memory became non‐significant when controlling for insomnia severity and depressive symptoms, the relationship held when controlling for objective sleep.

## 5 DISCUSSION

This exploratory study investigated the relationship between sleep discrepancy and cognitive function in community‐dwelling older adults. We found distinct groups characterised by differences in self‐reported sleep, objective sleep and/or sleep discrepancy across TST, WASO and SE. Interestingly, there were clusters characterised by a negative discrepancy identified across all sleep parameters, while an additional positive discrepancy cluster was found only for WASO. To our knowledge, this is the first study to use cluster analysis to characterise sleep discrepancy and quantity/quality simultaneously in a community‐dwelling sample of older adults.

### 5.1 Sleep discrepancy and cognition

Regarding negative discrepancy, there was no significant relationship between negative TST discrepancy and cognition, while analyses pertaining to WASO and SE showed that poorer delayed memory was associated with a greater likelihood of belonging to clusters characterised by a negative discrepancy, with medium effect sizes.

For WASO and SE, the negative discrepancy–poorer delayed memory association remained significant when controlling for objective sleep, with medium–large effect sizes. This suggests that the link between negative discrepancy and cognition may not simply be due to the deleterious neurobiological effects of poor objective sleep and its impact on processes such as memory consolidation (Rasch & Born, 2013).

For WASO, the relationship between negative discrepancy and delayed memory became non‐significant when controlling for insomnia severity and depressive symptoms, respectively. This suggests that the shared variance across insomnia severity, depressive symptoms and sleep discrepancy might underpin the association between negative WASO discrepancy and poorer delayed memory. This contrasts with findings for SE, where the relationship between poorer delayed memory and negative discrepancy stayed significant when controlling for insomnia severity and depressive symptoms, respectively, with medium effect sizes. This suggests a unique link between SE‐based negative discrepancy and poorer delayed memory, independent of adjacent constructs associated with both negative discrepancy (Rezaie et al., 2018; Van Den Berg et al., 2008) and memory (McDermott & Ebmeier, 2009; Wardle‐Pinkston et al., 2019).

As for why discrepancy in SE, but not other parameters such as TST and WASO, showed a unique relationship with delayed memory, the following logic may apply: estimating SE is relatively difficult and may be prone to over/underestimation, as it involves recall and summation of multiple pieces of information. Further, TIB, which distinguishes SE from TST, and is not included in the calculation of WASO, may play a role: a longer TIB could represent a greater degree of worry about sleep, and may be an important contributor to the sleep discrepancy–cognition relationship, although this is speculative.

With the variance in objective sleep removed, our post‐hoc findings suggest that the two remaining components of the clusters, self‐reported sleep and the difference score, are the driving factors behind the SE discrepancy–delayed memory relationship. A proximal explanation is that of a “placebo effect”: perception of sleep on a given night could lead to better or worse cognitive function the next day, depending on the direction (i.e. positive or negative) of sleep discrepancy (Draganich & Erdal, 2014).

Alternatively, perceived poor sleep could contribute to long‐term effects on cognition through allostatic load, the cumulative burden of chronic stress and its harmful effects on multiple physiological systems including the brain (McEwen, 1998); indeed, sleep disturbances have been linked to higher allostatic load (Christensen et al., 2022), which in turn has been linked to poorer cognition (D'Amico et al., 2020). Granted, allostatic load may also be involved in insomnia and depression (McEwen, 1998); it is possible that the relationships between insomnia, depression and sleep discrepancy with cognition are underpinned by allostatic load. This notion is consistent with the finding that the negative WASO discrepancy–poorer delayed memory association became non‐significant when controlling for insomnia severity and depressive symptoms. Nevertheless, it remains unclear to what extent each form of pathology is mediated by allostatic load, and the finding where negative SE discrepancy showed a unique relationship with poorer delayed memory outside of the effects of insomnia severity and depressive symptoms suggests a unique pathway between sleep discrepancy and cognitive impairment. Further, while allostatic load may be common to sleep discrepancy, insomnia and depression, the latter two conditions are associated with functional brain changes (Hwang & Kim, 2020; Kaiser et al., 2015); it is possible the same is true for sleep discrepancy, although this is yet to be investigated alongside functional neuroimaging.

The relationship between negative discrepancy in sleep quality and poorer delayed memory noted in the current study is partially consistent with previous research linking absolute (Van Den Berg et al., 2008; Winer et al., 2021), negative (Hita‐Yanez et al., 2013; Winer et al., 2021) and positive (DiNapoli et al., 2017; Most et al., 2012; Van Den Berg et al., 2008) discrepancy to poorer cognition. Together, this suggests that greater sleep discrepancy in either direction may be a risk factor for future cognitive decline. It should be acknowledged, however, that there may be a bi‐directional relationship between sleep discrepancy and cognition: poorer cognition could lead to inaccurate estimation of sleep, resulting in sleep discrepancy. Longitudinal studies are needed to investigate this possible bi‐directional relationship, along with mechanisms underpinning this relationship; the current study found no effects for attention and executive function, which may suggest that SE discrepancy is tracking early AD pathology, as this tends to prioritise regions involved in memory function (e.g. hippocampus).

### 5.2 Strengths, limitations and future research

A strength of the current study is the use of cluster analysis, which allowed comprehensive investigation of sleep discrepancy (i.e. clustering using self‐reported sleep, objective sleep and the difference score), accounting for some limitations of difference scores (Shanock et al., 2010). For example, it is possible for two individuals to arrive at the same difference score despite their sleep being characterised differently (e.g. 8 versus 4 hr of self‐reported/objective TST resulting in a difference score of 0 hr).

The use of sleep diary and actigraphy in the current study constituted a strength, as it allowed for measurement of discrepancy over multiple nights, across the same time period, of participants' habitual sleep. This holds an advantage over previous research assessing discrepancy using polysomnography (i.e. few nights in a novel environment) or questionnaires (i.e. may be more affected by recall bias/cognitive decline). However, actigraphy‐derived objective sleep estimates were constrained by self‐reported estimates based on sleep diary; self‐reported in‐bed and out‐of‐bed times were used to define the objective sleep period, potentially eliminating key variance in sleep discrepancy. Moreover, actigraphy may be prone to overestimating sleep, miscalculating lack of movement as sleep (Martin & Hakim, 2011), also affecting sleep discrepancy. To address these limitations, future research could use actigraphy devices with event marker buttons to indicate in‐bed and out‐of‐bed times, or ambulatory polysomnography.

Finally, because the current study was exploratory, adjustment for multiple comparisons was not performed (Althouse, 2016). We emphasise that our findings should be interpreted with caution, with any conclusions around the relationship between sleep discrepancy and memory in older adults pending independent replicability of our results. In saying this, the current study provides insight into the potential for cluster analysis in characterising sleep discrepancy, along with the specific sleep parameters and cognitive domains worth exploring. Indeed, given that medium–large effect sizes were found, further investigation of the sleep discrepancy–cognition relationship seems warranted.

## 6 CONCLUSION

This was the first study to explore the relationship of sleep discrepancy to cognitive function in community‐dwelling older adults using a cluster analytic approach. We found distinct clusters based on self‐reported and objectively‐measured sleep, and discrepancy, across various parameters (i.e. TST, WASO and SE). Poorer delayed memory was associated with negative WASO and SE discrepancy. Notably, the negative SE discrepancy–poorer delayed memory association remained significant when controlling for insomnia severity, depressive symptoms and objective sleep, suggesting a unique role of sleep discrepancy. Further investigation into the implications of negative and positive sleep discrepancy is warranted, particularly in older adults where sleep discrepancy and cognitive decline are evident. Ultimately, this line of research will serve to elucidate sleep discrepancy and its clinical utility.

## AUTHOR CONTRIBUTIONS

**Nadia Soh:** Conceptualization; writing – original draft; formal analysis; writing – review and editing; methodology; investigation; visualization. **Stephanie R. Rainey‐Smith:** Conceptualization; writing – review and editing; supervision. **James D. Doecke:** Formal analysis; methodology; writing – review and editing; supervision. **Rodrigo Canovas:** Formal analysis; methodology; writing – review and editing; supervision. **Romola S. Bucks:** Conceptualization; funding acquisition; writing – review and editing; supervision. **Melissa Ree:** Conceptualization; writing – review and editing; supervision. **Michael Weinborn:** Conceptualization; funding acquisition; writing – review and editing; supervision.

## CONFLICT OF INTEREST STATEMENT

NS is supported by an Australian Government Research Training Project (RTP) stipend. SRRS is supported by a National Health and Medical Research Council (NHMRC) Investigator Grant (GNT1197315).

## Supporting information


**DATA S1.** Supplementary information.

## Data Availability

The data that support the findings of this study are available from the corresponding author upon reasonable request.

## References

[jsr14288-bib-0001] Althouse, A. D. (2016). Adjust for multiple comparisons? It's not that simple. The Annals of Thoracic Surgery, 101(5), 1644–1645. 10.1016/j.athoracsur.2015.11.024 27106412

[jsr14288-bib-0002] Ancoli‐Israel, S. , Cole, R. , Alessi, C. , Chambers, M. , Moorcroft, W. , & Pollak, C. P. (2003). The role of actigraphy in the study of sleep and circadian rhythms. Sleep, 26(3), 342–392. 10.1093/sleep/26.3.342 12749557

[jsr14288-bib-0003] Ancoli‐Israel, S. , Martin, J. L. , Blackwell, T. , Buenaver, L. , Liu, L. , Meltzer, L. J. , Sadeh, A. , Spira, A. P. , & Taylor, D. J. (2015). The SBSM guide to actigraphy monitoring: Clinical and research applications. Behavioral Sleep Medicine, 13(Suppl 1), S4–S38. 10.1080/15402002.2015.1046356 26273913

[jsr14288-bib-0004] Baillet, M. , Cosin, C. , Schweitzer, P. , Pérès, K. , Catheline, G. , Swendsen, J. , & Mayo, W. (2016). Mood influences the concordance of subjective and objective measures of sleep duration in older adults. Frontiers in Aging Neuroscience, 8, 181. 10.3389/fnagi.2016.00181 27507944 PMC4960206

[jsr14288-bib-0005] Bastien, C. H. , Vallières, A. , & Morin, C. M. (2001). Validation of the insomnia severity index as an outcome measure for insomnia research. Sleep Medicine, 2(4), 297–307. 10.1016/s1389-9457(00)00065-4 11438246

[jsr14288-bib-0006] Benton, A. L. , Hamsher, d. S. K. , & Sivan, A. B. (1983). Controlled oral word association test (COWAT) [Database record]. APA PsycTests. 10.1037/t10132-000

[jsr14288-bib-0007] Carney, C. E. , Buysse, D. J. , Ancoli‐Israel, S. , Edinger, J. D. , Krystal, A. D. , Lichstein, K. L. , & Morin, C. M. (2012). The consensus sleep diary: Standardizing prospective sleep self‐monitoring. Sleep, 35(2), 287–302. 10.5665/sleep.1642 22294820 PMC3250369

[jsr14288-bib-0008] Charrad, M. , Ghazzali, N. , Boiteau, V. , & Niknafs, A. (2014). NbClust: An R package for determining the relevant number of clusters in a data set. Journal of Statistical Software, 61(6), 1–36. 10.18637/jss.v061.i06

[jsr14288-bib-0009] Christensen, D. S. , Zachariae, R. , Amidi, A. , & Wu, L. M. (2022). Sleep and allostatic load: A systematic review and meta‐analysis. Sleep Medicine Reviews, 64, 101650. 10.1016/j.smrv.2022.101650 35704985

[jsr14288-bib-0010] Cole, R. J. , Kripke, D. F. , Gruen, W. , Mullaney, D. J. , & Gillin, J. C. (1992). Automatic sleep/wake identification from wrist activity. Sleep, 15(5), 461–469. 10.1093/sleep/15.5.461 1455130

[jsr14288-bib-0011] D'Amico, D. , Amestoy, M. E. , & Fiocco, A. J. (2020). The association between allostatic load and cognitive function: A systematic and meta‐analytic review. Psychoneuroendocrinology, 121, 104849. 10.1016/j.psyneuen.2020.104849 32892066

[jsr14288-bib-0012] DiNapoli, E. A. , Gebara, M. A. , Kho, T. , Butters, M. A. , Gildengers, A. G. , Albert, S. M. , Dew, M. A. , Erickson, K. I. , Reynolds, C. F., III , & Karp, J. F. (2017). Subjective‐objective sleep discrepancy in older adults with MCI and subsyndromal depression. Journal of Geriatric Psychiatry and Neurology, 30(6), 316–323. 10.1177/0891988717731827 28954595 PMC5916761

[jsr14288-bib-0013] Draganich, C. , & Erdal, K. (2014). Placebo sleep affects cognitive functioning. Journal of Experimental Psychology. Learning, Memory, and Cognition, 40(3), 857–864. 10.1037/a0035546 24417326

[jsr14288-bib-0014] Dzierzewski, J. M. , Dautovich, N. , & Ravyts, S. (2018). Sleep and cognition in older adults. Sleep Medicine Clinics, 13(1), 93–106. 10.1016/j.jsmc.2017.09.009 29412987 PMC5841581

[jsr14288-bib-0015] Fisk, J. E. , & Sharp, C. A. (2004). Age‐related impairment in executive functioning: Updating, inhibition, shifting, and access. Journal of Clinical and Experimental Neuropsychology, 26(7), 874–890. 10.1080/13803390490510680 15742539

[jsr14288-bib-0016] Foley, D. J. , Monjan, A. A. , Brown, S. L. , Simonsick, E. M. , Wallace, R. B. , & Blazer, D. G. (1995). Sleep complaints among elderly persons: An epidemiologic study of three communities. Sleep, 18(6), 425–432. 10.1093/sleep/18.6.425 7481413

[jsr14288-bib-0017] Friedman, N. P. , & Miyake, A. (2017). Unity and diversity of executive functions: Individual differences as a window on cognitive structure. Cortex, 86, 186–204. 10.1016/j.cortex.2016.04.023 27251123 PMC5104682

[jsr14288-bib-0018] Hirshkowitz, M. , Whiton, K. , Albert, S. M. , Alessi, C. , Bruni, O. , DonCarlos, L. , Hazen, N. , Herman, J. , Hillard, P. J. A. , & Katz, E. S. (2015). National Sleep Foundation's updated sleep duration recommendations. Sleep Health, 1(4), 233–243. 10.1016/j.sleh.2015.10.004 29073398

[jsr14288-bib-0019] Hita‐Yanez, E. , Atienza, M. , & Cantero, J. L. (2013). Polysomnographic and subjective sleep markers of mild cognitive impairment. Sleep, 36(9), 1327–1334. 10.5665/sleep.2956 23997365 PMC3738041

[jsr14288-bib-0020] Hwang, Y. , & Kim, S.‐J. (2020). Brain activation changes in insomnia: A review of functional magnetic resonance imaging studies. Chronobiology in Medicine, 2(3), 103–108.

[jsr14288-bib-0021] Jackowska, M. , Dockray, S. , Hendrickx, H. , & Steptoe, A. (2011). Psychosocial factors and sleep efficiency: Discrepancies between subjective and objective evaluations of sleep. Psychosomatic Medicine, 73(9), 810–816. 22021463 10.1097/PSY.0b013e3182359e77

[jsr14288-bib-0022] Kaiser, R. H. , Andrews‐Hanna, J. R. , Wager, T. D. , & Pizzagalli, D. A. (2015). Large‐scale network dysfunction in major depressive disorder: A meta‐analysis of resting‐state functional connectivity. JAMA Psychiatry, 72(6), 603–611. 10.1001/jamapsychiatry.2015.0071 25785575 PMC4456260

[jsr14288-bib-0023] Kramer, J. H. , Mungas, D. , Possin, K. L. , Rankin, K. P. , Boxer, A. L. , Rosen, H. J. , Bostrom, A. , Sinha, L. , Berhel, A. , & Widmeyer, M. (2014). NIH EXAMINER: Conceptualization and development of an executive function battery. Journal of the International Neuropsychological Society, 20(1), 11–19. 10.1017/S1355617713001094 24103232 PMC4474183

[jsr14288-bib-0024] Kroenke, K. , Spitzer, R. L. , & Williams, J. B. (2001). The PHQ‐9: Validity of a brief depression severity measure. Journal of General Internal Medicine, 16(9), 606–613. 10.1046/j.1525-1497.2001.016009606.x 11556941 PMC1495268

[jsr14288-bib-0025] Littner, M. , Kushida, C. A. , Anderson, W. M. , Bailey, D. , Berry, R. B. , Davila, D. G. , Hirshkowitz, M. , Kapen, S. , Kramer, M. , & Loube, D. (2003). Practice parameters for the role of actigraphy in the study of sleep and circadian rhythms: An update for 2002. Sleep, 26(3), 337–341. 10.1093/sleep/26.3.337 12749556

[jsr14288-bib-0026] Manconi, M. , Ferri, R. , Sagrada, C. , Punjabi, N. M. , Tettamanzi, E. , Zucconi, M. , Oldani, A. , Castronovo, V. , & Ferini‐Strambi, L. (2010). Measuring the error in sleep estimation in normal subjects and in patients with insomnia. Journal of Sleep Research, 19(3), 478–486. 10.1111/j.1365-2869.2009.00801.x 20149068

[jsr14288-bib-0027] Martin, J. L. , & Hakim, A. D. (2011). Wrist actigraphy. Chest, 139(6), 1514–1527. 10.1378/chest.10-1872 21652563 PMC3109647

[jsr14288-bib-0028] McDermott, L. M. , & Ebmeier, K. P. (2009). A meta‐analysis of depression severity and cognitive function. Journal of Affective Disorders, 119(1–3), 1–8. 10.1016/j.jad.2009.04.022 19428120

[jsr14288-bib-0029] McEwen, B. S. (1998). Stress, adaptation, and disease. Allostasis and allostatic load. Annals of the new York Academy of Sciences, 840, 33–44. 10.1111/j.1749-6632.1998.tb09546.x 9629234

[jsr14288-bib-0030] Most, E. I. , Aboudan, S. , Scheltens, P. , & Van Someren, E. J. (2012). Discrepancy between subjective and objective sleep disturbances in early‐and moderate‐stage Alzheimer disease. The American Journal of Geriatric Psychiatry, 20(6), 460–467. 10.1097/JGP.0b013e318252e3ff 22531105

[jsr14288-bib-0031] Netzer, N. C. , Stoohs, R. A. , Netzer, C. M. , Clark, K. , & Strohl, K. P. (1999). Using the Berlin questionnaire to identify patients at risk for the sleep apnea syndrome. Annals of Internal Medicine, 131(7), 485–491. 10.7326/0003-4819-131-7-199910050-00002 10507956

[jsr14288-bib-0032] Ohayon, M. , Wickwire, E. M. , Hirshkowitz, M. , Albert, S. M. , Avidan, A. , Daly, F. J. , Dauvilliers, Y. , Ferri, R. , Fung, C. , & Gozal, D. (2017). National Sleep Foundation's sleep quality recommendations: First report. Sleep Health, 3(1), 6–19. 10.1016/j.sleh.2016.11.006 28346153

[jsr14288-bib-0033] R Core Team . (2023). R: A language and environment for statistical computing. R Foundation for Statistical Computing. https://www.R-project.org/

[jsr14288-bib-0034] Randolph, C. , Tierney, M. C. , Mohr, E. , & Chase, T. N. (1998). The repeatable battery for the assessment of neuropsychological status (RBANS): Preliminary clinical validity. Journal of Clinical and Experimental Neuropsychology, 20(3), 310–319. 10.1076/jcen.20.3.310.823 9845158

[jsr14288-bib-0035] Rasch, B. , & Born, J. (2013). About sleep's role in memory. Physiological Reviews, 93(2), 681–766. 10.1152/physrev.00032.2012 23589831 PMC3768102

[jsr14288-bib-0036] Reitan, R. M. (1958). Validity of the trail making test as an indicator of organic brain damage. Perceptual and Motor Skills, 8(3), 271–276. 10.2466/pms.1958.8.3.271

[jsr14288-bib-0037] Rezaie, L. , Fobian, A. D. , McCall, W. V. , & Khazaie, H. (2018). Paradoxical insomnia and subjective–objective sleep discrepancy: A review. Sleep Medicine Reviews, 40, 196–202. 10.1016/j.smrv.2018.01.002 29402512

[jsr14288-bib-0038] Rotenberg, V. S. , Indursky, P. , Kayumov, L. , Sirota, P. , & Melamed, Y. (2000). The relationship between subjective sleep estimation and objective sleep variables in depressed patients. International Journal of Psychophysiology, 37(3), 291–297. 10.1016/s0167-8760(00)00110-0 10858574

[jsr14288-bib-0039] Shanock, L. R. , Baran, B. E. , Gentry, W. A. , Pattison, S. C. , & Heggestad, E. D. (2010). Polynomial regression with response surface analysis: A powerful approach for examining moderation and overcoming limitations of difference scores. Journal of Business and Psychology, 25(4), 543–554. 10.1007/s10869-010-9183-4

[jsr14288-bib-0040] Staner, L. (2010). Comorbidity of insomnia and depression. Sleep Medicine Reviews, 14(1), 35–46. 10.1016/j.smrv.2009.09.003 19939713

[jsr14288-bib-0041] Strauss, E. , Sherman, E. M. S. , & Spreen, O. (2006). A compendium of neuropsychological tests: Administration, norms, and commentary (3rd ed.). Oxford University Press.

[jsr14288-bib-0042] Utsumi, T. , Yoshiike, T. , Kaneita, Y. , Aritake‐Okada, S. , Matsui, K. , Nagao, K. , Saitoh, K. , Otsuki, R. , Shigeta, M. , & Suzuki, M. (2022). The association between subjective–objective discrepancies in sleep duration and mortality in older men. Scientific Reports, 12(1), 18650. 10.1038/s41598-022-22065-8 36333394 PMC9636161

[jsr14288-bib-0043] van Buuren, S. , & Groothuis‐Oudshoorn, K. (2011). Mice: Multivariate imputation by chained equations in R. Journal of Statistical Software, 45(3), 1–67. 10.18637/jss.v045.i03

[jsr14288-bib-0044] Van Den Berg, J. F. , Van Rooij, F. J. , Vos, H. , Tulen, J. H. , Hofman, A. , Miedema, H. M. , Neven, A. K. , & Tiemeier, H. (2008). Disagreement between subjective and actigraphic measures of sleep duration in a population‐based study of elderly persons. Journal of Sleep Research, 17(3), 295–302. 10.1111/j.1365-2869.2008.00638.x 18321246

[jsr14288-bib-0045] Venables, W. , & Ripley, B. (2002). Modern applied statistics with S (4th ed.). Springer.

[jsr14288-bib-0046] Wardle‐Pinkston, S. , Slavish, D. C. , & Taylor, D. J. (2019). Insomnia and cognitive performance: A systematic review and meta‐analysis. Sleep Medicine Reviews, 48, 101205. 10.1016/j.smrv.2019.07.008 31522135

[jsr14288-bib-0047] Wechsler, D. (1997). Wechsler adult intelligence scale–Third edition (WAIS‐III) [Database record]. APA PsycTests. 10.1037/t49755-000

[jsr14288-bib-0048] Winer, J. R. , Morehouse, A. , Fenton, L. , Harrison, T. M. , Ayangma, L. , Reed, M. , Kumar, S. , Baker, S. L. , Jagust, W. J. , & Walker, M. P. (2021). Tau and β‐amyloid burden predict actigraphy‐measured and self‐reported impairment and misperception of human sleep. Journal of Neuroscience, 41(36), 7687–7696. 10.1523/JNEUROSCI.0353-21.2021 34290080 PMC8425979

[jsr14288-bib-0049] Winser, M. A. , McBean, A. L. , & Montgomery‐Downs, H. E. (2013). Minimum duration of actigraphy‐defined nocturnal awakenings necessary for morning recall. Sleep Medicine, 14(7), 688–691. 10.1016/j.sleep.2013.03.018 23746600

[jsr14288-bib-0050] Yoon, G. , Lee, M. H. , Oh, S. M. , Choi, J.‐W. , Yoon, S. Y. , & Lee, Y. J. (2022). Negative and positive sleep state misperception in patients with insomnia: Factors associated with sleep perception. Journal of Clinical Sleep Medicine, 18(7), 1789–1795. 10.5664/jcsm.9974 35383568 PMC9243288

