# Supplementary material for: Sleep discrepancy and cognitive function in community‐dwelling older adults
Source: J Sleep Res. 2024 Jul 26;34(1):e14288. doi: 10.1111/jsr.14288 (PMC11744247; doi:10.1111/jsr.14288)
Supplement: Supplementary file 1 — DATA S1. Supplementary information. [file JSR-34-e14288-s001.docx]

**Supplementary Material**

Table S.1.1.

*2x2 contingency table for Self-Reported TST*

|  | *Cluster 1* | *Cluster 2* |
| --- | --- | --- |
| < 360 minutes | 4 | 35 |
| > 360 minutes | 170 | 7 |

*Note.* *N* = 216. Cluster 1 = “TST 1: no sleep discrepancy and longer sleep duration”; Cluster 2 = “TST 2: negative sleep discrepancy and shorter sleep duration”. TST = Total Sleep Time (minutes).

Table S.1.2.

*2x2 contingency table for Objective TST*

|  | *Cluster 1* | *Cluster 2* |
| --- | --- | --- |
| < 360 minutes | 8 | 13 |
| > 360 minutes | 166 | 26 |

*Note.* *N* = 216. Cluster 1 = “TST 1: no sleep discrepancy and longer sleep duration”; Cluster 2 = “TST 2: negative sleep discrepancy and shorter sleep duration”. TST = Total Sleep Time (minutes).

Table S.1.3.

*2x2 contingency table for TST Discrepancy*

|  | *Cluster 1* | *Cluster 2* |
| --- | --- | --- |
| < 0 minutes | 82 | 41 |
| > 0 minutes | 92 | 1 |

*Note.* *N* = 216. Cluster 1 = “TST 1: no sleep discrepancy and longer sleep duration”; Cluster 2 = “TST 2: negative sleep discrepancy and shorter sleep duration”. TST = Total Sleep Time (minutes).

Table S.2.1.

*3x2 contingency table for Self-Reported WASO*

|  | *Cluster 1* | *Cluster 2* | *Cluster 3* |
| --- | --- | --- | --- |
| > 30 minutes | 35 | 39 | 7 |
| < 30 minutes | 105 | 0 | 29 |

*Note.* *N* = 215. Cluster 1 = “WASO 1: no sleep discrepancy and good sleep quality”; Cluster 2 = “WASO 2: negative sleep discrepancy and poor sleep quality”; Cluster 3 = “WASO 3: positive sleep discrepancy and poor sleep quality”. WASO = Wake After Sleep Onset (minutes).

Table S.2.2.

*3x2 contingency table for Objective WASO*

|  | *Cluster 1* | *Cluster 2* | *Cluster 3* |
| --- | --- | --- | --- |
| > 30 minutes | 70 | 32 | 36 |
| < 30 minutes | 70 | 7 | 0 |

*Note.* *N* = 215. Cluster 1 = “WASO 1: no sleep discrepancy and good sleep quality”; Cluster 2 = “WASO 2: negative sleep discrepancy and poor sleep quality”; Cluster 3 = “WASO 3: positive sleep discrepancy and poor sleep quality”. WASO = Wake After Sleep Onset (minutes).

Table S.2.3.

*3x2 contingency table for WASO Discrepancy*

|  | *Cluster 1* | *Cluster 2* | *Cluster 3* |
| --- | --- | --- | --- |
| > 0 minutes | 25 | 36 | 0 |
| < 0 minutes | 115 | 3 | 36 |

*Note.* *N* = 215. Cluster 1 = “WASO 1: no sleep discrepancy and good sleep quality”; Cluster 2 = “WASO 2: negative sleep discrepancy and poor sleep quality”; Cluster 3 = “WASO 3: positive sleep discrepancy and poor sleep quality”. WASO = Wake After Sleep Onset (minutes).

Table S.3.1.

*2x2 contingency table for Self-Reported SE*

|  | *Cluster 1* | *Cluster 2* |
| --- | --- | --- |
| < 80% | 18 | 88 |
| > 80% | 104 | 5 |

*Note.* *N* = 215. Cluster 1 = “SE 1: no sleep discrepancy and good self-reported sleep quality”. Cluster 2 = “SE 2: negative discrepancy and poor self-reported sleep quality”; SE = Sleep Efficiency (%).

Table S.3.2.

*2x2 contingency table for Objective SE*

|  | *Cluster 1* | *Cluster 2* |
| --- | --- | --- |
| < 80% | 3 | 0 |
| > 80% | 119 | 93 |

*Note.* *N* = 215. Cluster 1 = “SE 1: no sleep discrepancy and good self-reported sleep quality”. Cluster 2 = “SE 2: negative sleep discrepancy and poor self-reported sleep quality”; SE = Sleep Efficiency (%).

Table S.3.3.

*2x2 contingency table for SE Discrepancy*

|  | *Cluster 1* | *Cluster 2* |
| --- | --- | --- |
| < 0% | 90 | 93 |
| > 0% | 32 | 0 |

*Note.* *N* = 215. Cluster 1 = “SE 1: no sleep discrepancy and good self-reported sleep quality”. Cluster 2 = “SE 2: negative sleep discrepancy and poor self-reported sleep quality”; SE = Sleep Efficiency (%).
